# Prediction of menstrual patterns and analysis of adverse effects for hysteroscopic endometrial polypectomy combined with LNG-IUS treatment: a single-center retrospective cohort study

**DOI:** 10.1186/s40001-025-03776-w

**Published:** 2026-01-05

**Authors:** Ruikun Zhao, Yinan Chen, Ping Lu, Yuanyuan Hu, Quanjia Jiang, Qin Zhou

**Affiliations:** 1 Department of Obstetrics and Gynecology, The First Affiliated Hospital of Chongqing Medical University, No.1 Youyi Road, Yuzhong District, Chongqing, 400016 China; 2 Department of Mathematics, School of Mathematics and Physics, University College London, London, WC1E 6AE UK; 3 Department of Obstetrics and Gynecology, Chongqing Shapingba Maternity and Child Healthcare Hospital, Shapingba District, Chongqing, 401331 China

**Keywords:** Endometrial polyp, Levonorgestrel-releasing Intrauterine System (LNG-IUS), Amenorrhea, Irregular Uterine Bleeding Pattern (IUBP), Placement timing, Generalized Additive Model (GAM), Adverse effect

## Abstract

**Background:**

This study aimed to analyze the clinical data within one year of the patients who underwent hysteroscopic endometrial polypectomy with levonorgestrel-releasing intrauterine system (LNG-IUS) insertion, and to identify the risk factors affecting the duration of irregular uterine bleeding pattern (IUBP), trying to build models predicting this period and evaluate the performance of the models, in order to shorten the IUBP duration through intervention.

**Methods:**

Clinical data were collected from 245 patients who underwent hysteroscopic endometrial polypectomy and LNG-IUS placement at our hospital between January 2018 and December 2022. The data, recorded within one year after LNG-IUS insertion, included age, the number of polyps, preoperative endometrial thickness, maximum polyp diameter, the timing of LNG-IUS placement, LNG-IUS expulsion and migration, coital bleeding, pelvic pain, amenorrhea, irregular uterine bleeding pattern, and polyp recurrence. The differences of adverse reactions were analyzed by age and LNG-IUS placement timing stratified. Logistic regression model and Generalized Additive Model (GAM) were established to predict the duration of irregular uterine bleeding after LNG-IUS insertion, and attempts were made to explore the relationship between the variables.

**Results:**

Age was the independent risk factor for amenorrhea after LNG-IUS insertion, the patients with aged ≥ 40 years were more likely to experience amenorrhea (P < 0.05). No significant difference was observed between patients who had LNG-IUS placement at surgery immediately and those who had LNG-IUS placement within 3 months postoperatively regarding the incidence of adverse reactions. Preoperative endometrial thickness and maximum polyp diameter were the independent risk factors for the duration of irregular uterine bleeding after LNG-IUS insertion. Comprised preoperative endometrial thickness and maximum polyp diameter identified using logistic regression model and GAM model could predict the duration of IUBP effectively. The tenfold cross-validation showed that the GAM model (AUC 0.906) had slightly better predictive power than the bivariate logistic regression model (AUC 0.902).

**Conclusions:**

Hysteroscopic polypectomy combined with LNG-IUS insertion was an effective measure to treat endometrial polyps. LNG-IUS intraoperative placement did not increase the incidence of adverse reactions. Preoperative endometrial thickness and maximum polyp diameter play a key role in valuable prediction for the duration of irregular uterine bleeding after LNG-IUS insertion.

**Supplementary Information:**

The online version contains supplementary material available at 10.1186/s40001-025-03776-w.

## Background

Endometrial polyp is the common benign intrauterine lesion, often leading to abnormal uterine bleeding and infertility, although it may also be asymptomatic [1]. Transvaginal ultrasound (TVUS) is currently the primary method for diagnosing endometrial polyp [2]. Hysteroscopic endometrial polypectomy remains the gold standard for treatment, but the recurrence rate after surgery is relatively high, ranging from 2.5% to 43.6%, the higher number of endometrial polyps and the longer follow-up duration are associated with the greater potential of polyp recurrence [3]. To prevent the recurrence of endometrial polyp, progestins, oral contraceptives can be recommended in clinic. The LNG-IUS, though primarily a contraceptive device, offers considerable therapeutic benefits for gynecological conditions including heavy menstrual bleeding, adenomyosis, endometrial hyperplasia, dysmenorrhea, and endometrial polyps. This therapeutic application for treating endometrial polyps is further supported by multiple expert consensus guidelines in China [4, 5]. Several studies indicate that, compared to oral progesterone, hysteroscopic polypectomy combined with a levonorgestrel-releasing intrauterine system (LNG-IUS) can significantly reduce the risk of endometrial polyp recurrence [6–8].

However, unpredictable uterine bleeding following LNG-IUS placement is a common clinical concern and a primary reason for patient anxiety. This is also the main reason why patients refuse to accept LNG-IUS placement for the purpose of preventing polyp recurrence. In this study, we defined Irregular Uterine Bleeding Pattern (IUBP) as non-cyclical or unscheduled spotting occurring after LNG-IUS placement for this purpose. It was characterized by either an intermenstrual interval of less than 21 days or a bleeding duration exceeding 8 days [9–11]. Some surgeons also express concern that intraoperative placement may increase the risk of intrauterine infection or device expulsion. Moreover, a few studies have directly compared adverse reactions between intraoperative and postoperative placement timing. Therefore, we analyzed the clinical characteristics of patients undergoing hysteroscopic polypectomy with LNG-IUS placement within one year. Our objectives were threefold: first, to contrast the adverse reaction profiles between the two placement timings; second, to identify risk factors affecting the duration of IUBP; and third, to develop predictive models for the IUBP duration. Ultimately, we aim to provide valuable strategies for reducing adverse reactions through targeted interventions.

## Methods

### Study design and data collection

This single-center retrospective cohort study analyzed data from a tertiary hospital in China. This study was approved by the Ethics Committee of the First Affiliated Hospital of Chongqing Medical University (CY2024-592–02). In our practice, hysteroscopic procedures are typically performed under non-intubated general anesthesia following a "see-and-treat" pathway. Consequently, detailed preoperative counseling is routinely conducted, covering the surgical approach, potential risks, and postoperative management strategies [3, 4].

Women eligible for inclusion were premenopausal individuals aged 18 years or older who were diagnosed with endometrial polyps and subsequently underwent hysteroscopic polypectomy between January 2018 and December 2022, all confirmed by postoperative histopathology. Additional inclusion criteria consisted of no previous hormonal therapy within three months before surgery, a regular preoperative menstrual cycle, LNG-IUS insertion either during the surgery or within three months postoperatively, absence of cervical lesions (ruled out by routine preoperative gynecologic examination), and no severe systemic diseases. Cases with amenorrhea older than 40 years of age underwent endocrine hormonal examination one year after LNG-IUS insertion to exclude menopause.

The exclusion criteria were as follows: removal of the LNG-IUS within one year for personal reasons (such as intolerance to irregular uterine bleeding, device expulsion, or pregnancy planning); endometrial hyperplasia confirmed by postoperative histopathology; use of hormonal therapy during the follow-up period; loss to follow-up within one year; and amenorrhea due to menopause (confirmed by endocrine hormonal examination).

Data were retrospectively gathered through a review of electronic medical records, outpatient follow-ups, and telephone interviews. From the electronic medical records, we extracted preoperative and surgical data, including patient age at the time of surgery, transvaginal ultrasound (TVUS) parameters, such as endometrial thickness and maximum polyp diameter, the number of polyps (recorded as single or multiple during hysteroscopic polypectomy), and the timing of LNG-IUS placement (categorized as intraoperative or postoperative within 3 months). Transvaginal ultrasound (TVUS) is scheduled for the follicular phase after menstruation, using the same model of equipment (GE VOLUSON E8, USA), and is performed by two ultrasound physicians with standardized training. If necessary, the examination is reviewed by a senior physician. Outcome data, assessed during outpatient follow-up and telephone interviews, included LNG-IUS expulsion and migration, coital bleeding, pelvic pain, amenorrhea, the duration of IUBP, and polyp recurrence. To minimize subjective variability in bleeding assessment, we scheduled outpatient follow-up every 3 months. At each visit, we used the pictorial blood loss assessment chart (PBAC) to objectively quantify menstrual blood loss over the preceding three-month period. The PBAC, a validated scoring system based on the number and saturation of soiled sanitary items, is detailed in Additional file 2: Figure S5. Finally, to address potential bias in the analysis, we utilized non-parametric tests and ordered logistic regression, acknowledging that while these methods mitigate the impact of bias, they cannot eliminate it entirely.

### Statistical analysis and model exploration

Descriptive analyses and inferential tests were conducted using Python (version 3.12.4). Categorical variables were compared using the Chi-square or Fisher’s exact test, as appropriate. Continuous variables were analyzed using the Mann–Whitney U test. To identify independent risk factors, multivariate logistic regression was employed. The discriminatory ability of the model was assessed by generating receiver operating characteristic (ROC) curves and calculating the corresponding area under the curve (AUC) values. Continuous variables are presented as median and interquartile range, while categorical variables are expressed as frequency (N) and percentage (%).

In this study, irregular uterine bleeding patterns (IUBP) following LNG-IUS placement was a common symptom. We classified its duration as a binary dependent variable based on whether it exceeded 3 months. This cutoff was selected based on expert consensus [11], as it effectively stratified the cohort into clinically distinct groups while aligning with our 3-month follow-up intervals, thereby ensuring assessment accuracy and serving as an early indicator for clinical management.

To predict the duration of IUBP after LNG-IUS placement, we built a model based on five clinical characteristics: age, number of polyps, LNG-IUS placement timing, preoperative endometrial thickness, and maximum polyp diameter. We strategically focused on including relevant preoperative factors. These core, objectively measurable factors are related to the polyp anatomy and the intervention and are routinely available in clinical practice. Furthermore, we sought to minimize confounding by implementing strict exclusion criteria regarding factors such as hormonal therapy during the follow-up period and endometrial hyperplasia confirmed by postoperative histopathology.

Initially, we employed univariate logistic regression analysis between variables and the duration of IUBP, determining the coefficient of each variable in the model. Subsequently, we used two logistic regression models, including full variable model and significant variable model. Additionally, to account for potentially non-linear relationships, a Generalized Additive Model (GAM) was employed to overcome the limitations of the linear assumption in conventional regression models. We found the configurations that provided the best performance and tenfold cross-validation were used in logistic regression models and GAM model. The diagnostic efficiency was verified by the ROC curve, area under the ROC curve (AUC), sensitivity, and specificity. The 95% confidence intervals for AUC were calculated using the bootstrap method. The model analysis was conducted using R version 4.4.2 (2024–10–31 ucrt) within RStudio 2024.12.0.467 on Windows 11 × 64. A two-sided significance level of P < 0.05 was considered statistically significant.

## Result

**Fig. 1:**
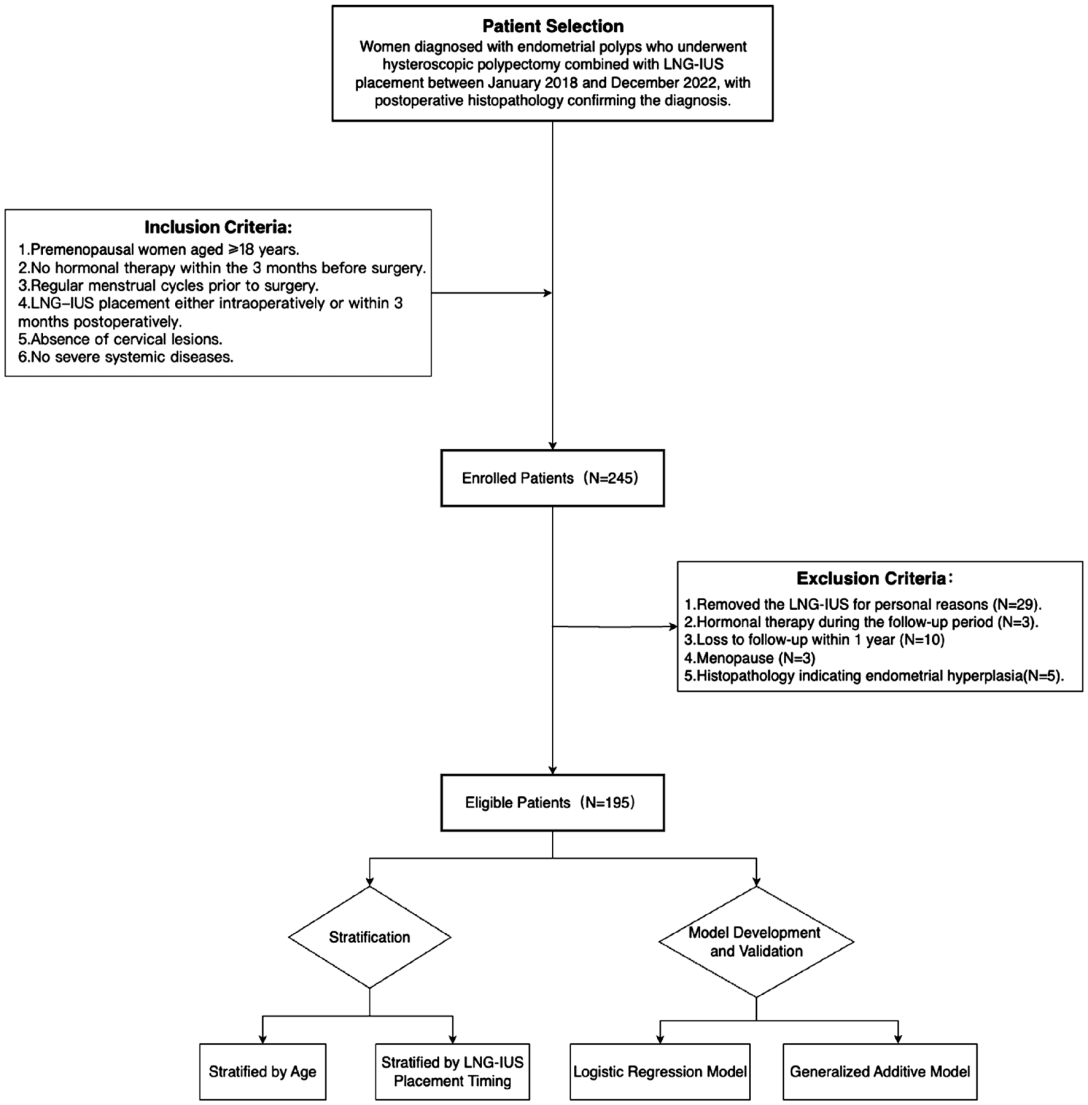
Flow chart

After screening among 245 candidates, 195 patients were included in the final analysis. A total of 50 participants were excluded based on the exclusion criteria. Specifically, twenty-nine cases were excluded due to removal of the LNG-IUS for personal reasons, the reasons were intolerance to irregular uterine bleeding (n = 17), device expulsion (n = 9), and pregnancy planning (n = 3). Other exclusions included the use of hormonal therapy during follow-up (n = 3), loss to follow-up within one year (n = 10), and postoperative histopathology confirming endometrial hyperplasia (n = 5). Additionally, three patients over the age of 40 who developed amenorrhea were excluded after endocrine hormonal examination confirmed natural menopause. They were advised to have the LNG-IUS removed. The flow chart of this study is shown in Fig. 1. The patients’ clinical characteristics are listed in Table 1. Whether grouped by age group or the LNG-IUS placement timing, there were no statistically significant differences in preoperative endometrial thickness and the maximum polyp diameter between the two groups (p > 0.05). In terms of adverse reactions (LNG-IUS expulsion and migration, coital bleeding, pelvic pain, amenorrhea, irregular uterine bleeding, and polyp recurrence) after LNG-IUS insertion, irregular uterine bleeding was a common concomitant symptom (100%), approximately 45.64% of cases had lasted beyond 3 months after LNG-IUS insertion, followed by pelvic pain (26.67%), amenorrhea (22.56%), and coital bleeding (22.05%). The occurrence of LNG-IUS expelled and migrate(11.79%) within one year of placement was less common, and polyp recurrence (2.05%) was rare.

**Table 1:** Description of participants’ characteristics and stratified analysis by age and LNG-IUS placement timing

Variables	All *N* = 195	Stratified Analysis by Age			Stratified Analysis by LNG-IUS placement timing		
		≥ 40 (n = 96)	< 40 (n = 99)	P-value	Intraoperative placement (n = 90)	Postoperative placement (n = 105)	P-value
Age (years)	38.96 ± 6.80				38.97 ± 6.81	38.96 ± 6.82	0.9067
Maximum polyp diameter (mm)	12.55 ± 6.68	12.54 ± 6.29	12.56 ± 7.07	0.71256	13.43 ± 7.06	11.79 ± 6.28	0.0545
Endometrial thickness (mm)	11.43 ± 3.31	11.52 ± 3.48	11.34 ± 3.14	0.73043	11.53 ± 3.08	11.34 ± 3.51	0.6831
LNG-IUS Placement timing							
Intraoperative placement	90(46.15%)	47(52.22%)	43(47.78%)	0.52874			
Postoperative placement within 3 months	105(53.85%)	49(46.67%)	56(53.33%)				
Number of Polyps							
Single polyp	92(47.18%)	50(54.35%)	42(45.65%)	0.2273	45 (50.00%)	47(44.76%)	0.558
Multiple polyps	103(52.82%)	46(44.66%)	57(55.34%)		45 (50.00%)	58(55.24%)	
LNG-IUS migration							
No	172(88.21%)	88 (91.70%)	84 (84.80%)	0.20995	81(90.00%)	91(86.70%)	0.619
Yes	23(11.79%)	8 (8.30%)	15 (15.20%)		9(10.00%)	14(13.30%)	
Coital bleeding							
No	152(77.95%)	78(81.25%)	74 (74.75%)	0.35642	72 (80.00%)	80 (76.19%)	0.641
Yes	43(22.05%)	18 (18.75%)	25 (25.25%)		18 (20.00%)	25 (23.81%)	
Pelvic pain							
No	143(73.33%)	77 (80.21%)	66 (66.67%)	0.04817	68 (75.56%)	75 (71.43%)	0.626
Yes	52(26.67%)	19 (19.79%)	33 (33.33%)		22 (24.44%)	30 (28.57%)	
Amenorrhea							
No	151(77.44%)	66(68.75%)	85(85.86%)	0.00723	71 (78.89%)	80 (76.19%)	0.781
Yes	44(22.56%)	30(31.25%)	14(14.14%)		19 (21.11%)	25 (23.81%)	
Polyp recurrence							
No	191(97.95%)	95(98.96%)	96(96.97%)	0.63538	88(97.78%)	103(98.10%)	1.000
Yes	4(2.05%)	1(1.04%)	3(3.03%)		2(2.22%)	2(1.90%)	
IUBP duration							
≤ 3 months	106(54.36%)	55(57.30%)	51(51.50%)	0.50551	49 (54.44%)	57 (54.29%)	1.000
> 3 months	89(45.64%)	41(42.70%)	48(48.50%)		41 (45.56%)	48 (45.71%)	

### Adverse reactions analyzed by age and LNG-IUS placement timing stratification

Based on the age distribution of the patients, they were divided into two groups, 99 (50.77%) cases aged less than 40 years old, and 96 (49.23%) cases aged 40 years or older. There was no significant difference in preoperative characteristics (P > 0.05). The adverse reactions were followed up after LNG-IUS insertion within one year. The incidence of pelvic pain in the younger group was higher than that in the older group (P < 0.05). The proportion of participants experiencing amenorrhea was significantly higher in the older group than in the younger group (P < 0.01). All the results are indicated in Table 1.

According to LNG-IUS placement timing, the patients were divided into two groups, 90 (46.15%) cases placed LNG-IUS in surgery and 105 (53.85%) cases inserted LNG-IUS within 3 months after surgery. This timing was determined by patient preference following preoperative counseling, with some opting for immediate insertion while others preferred to await final histopathological results. To assess potential selection bias, we compared baseline characteristics between the groups and found no significant differences in preoperative parameters (P > 0.05). Furthermore, no significant differences were observed between the two groups regarding LNG-IUS migration, coital bleeding, pelvic pain, amenorrhea, polyp recurrence, or duration of IUBP (P > 0.05). The comparison results of the two groups are shown in Table 1.

### Screening of risk formula in IUBP duration

**Fig. 2:**
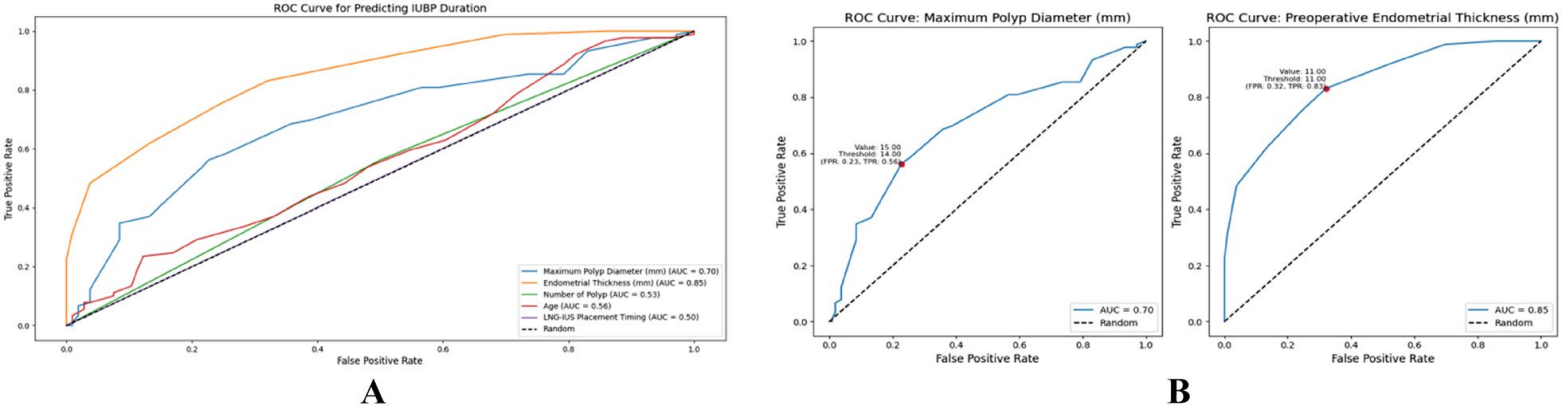
ROC Curve of the Univariate Logistic Regression Model for Predicting IUBP Duration. **A**: ROC Curve of five independent risk factors; **B** ROC Curve of Maximum Polyp Diameter and Endometrial Thickness (mm)

We explored the distribution of the five variables—age, preoperative endometrial thickness, maximum polyp diameter, number of polyps, and LNG-IUS placement timing—and found that the three continuous variables were not normally distributed (Additional file 1_Table S1). There were significant differences in endometrial thickness and maximum polyp diameter between two IUBP duration groups (Table 2, Additional file 2_FigureS1). Univariate logistic regression model also showed these two variables were the risk factors influencing IUBP duration following LNG-IUS placement (Table 3). The predictive ability of each variable was analyzed using ROC curves (Fig. 2-A). It showed that the AUC value for maximum polyp diameter and endometrial thickness in predicting IUBP duration were 0.696 and 0.848, respectively. When the endometrial thickness was less than 11 mm, there was an 83.1% probability that the IUBP duration would be less than 3 months after LNG-IUS insertion. When the maximum polyp diameter was less than 14 mm, there was a 56.2% probability that the IUBP duration would be less than 3 months.

**Table 2:** The variable distribution of different IUBP duration

Variable			IUBP duration		p-value
			** ≤ 3 months**	** > 3 months**	
Continuous variable	Age (Median ± IQR)(years)		40.00 ± 6.71	39.00 ± 6.85	0.171
	Endometrial thickness (Median ± IQR)(mm)		10.00 ± 2.38	13.00 ± 2.98	0.000
	Maximum polyp diameter (Median ± IQR)(mm)		10.00 ± 6.30	15.00 ± 6.54	0.000
Categorical variable	Number of Polyps	Single polyp	53(57.6%)	39(42.4%)	0.473
		Multiple polyps	53(51.5%)	50(48.5%)	
	LNG-IUS Placement timing	Postoperative placement within 3 months	57(53.8%)	49(46.2%)	1.000
		Intraoperative placement	48(53.9%)	41(46.1%)

**Table 3:** Univariate regression analysis of factors influencing IUBP duration

Variable	P-value	AUC	Cut-off	Youden's index	Sensitivity	Specificity
Maximum polyp diameter (mm)	< 0.001	0.696	14	0.335	0.562	0.774
Endometrial thickness (mm)	< 0.001	0.848	11	0.511	0.831	0.679
Age(years)	0.120	0.443	52	0.011	0.011	1.000
Number of Polyps	0.390	0.531	1	0.062	0.562	0.500
LNG-IUS Placement Timing	0.982	0.499	inf	0.000	0.000	1.000

### Models prediction IUBP duration following LNG-IUS placement and validation

**Fig. 3:**
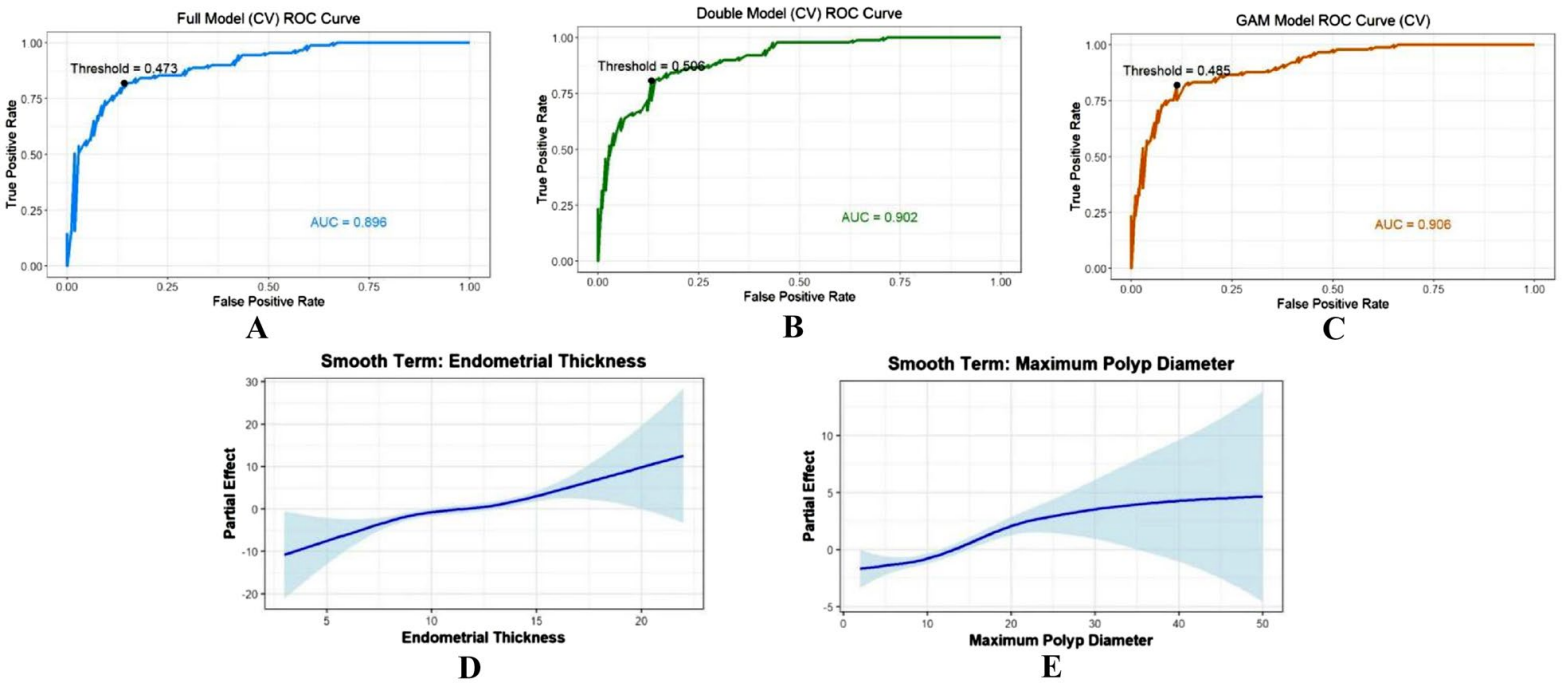
Full model, Double model and GAM model ROC Curve for Predicting IUBP Duration and The smooth curve of Endometrial Thickness and Maximum Polyp Diameter for GAM Model. **A**: Full Model ROC Curve for Predicting IUBP Duration by Cross-Validation; **B**: Double Model ROC Curve for Predicting IUBP Duration by Cross-Validation. **C**: GAM Model ROC Curve for Predicting IUBP Duration by Cross-Validation. **D** The smooth curve of Endometrial Thickness for GAM Model; **E** The smooth curve of Maximum Polyp Diameter for GAM Model

We performed two logistic regression models to evaluate the significance of the pre-surgery variables and the overall performance of the models. In Full model, it identified maximum polyp diameter (P < 0.01) and endometrial thickness (P < 0.01) as statistically significant predictors, whereas age, LNG-IUS placement timing, and number of polyps were not significant (Table 4). This model achieved an AIC of 157.03 and an AUC of 0.896, the accuracy of confusion matrix remained about 0.841 by tenfold cross-validation (Fig. 3-A, Table 5). Removing three invalid variables, a simplified Double model including endometrial thickness and maximum polyp diameter was constructed. It suggested that both variables are significant (p < 0.01), and the odds ratio (OR) of is greater than 1 (Table 4). This model achieved an AIC of 154.41 and an AUC of 0.902, the accuracy of confusion matrix remained about 0.841 by tenfold cross-validation (Fig. 3-B, Table 5). ANOVA test was used to compare the goodness-of-fit of the two logistic regression models. It indicated the goodness-of-fit difference between Double model and Full model was not significant (P = 0.336).

**Table 4:** Multivariate logistic regression of full model and double model for IUBP duration

Model	Variable	Estimate	Estimate 95% CI	Std. Error	z value	P value	OR
Full model	Intercept	− 9.274	–	1.927	− 4.812	< 0.001	
	Age	− 0.047	0.130–0.024	0.033	− 1.444	0.149	0.954
	Maximum Polyp Diameter (mm)	0.197	0.131–0.357	0.040	4.971	< 0.001	1.217
	Endometrial Thickness (mm)	0.741	0.598–1.049	0.108	6.879	< 0.001	2.098
	LNG-IUS Placement Timing	− 0.346	− 1.286–0.475	0.423	− 0.819	0.413	0.707
	Number of Polyps	0.273	− 0.658–1.231	0.425	0.642	0.521	1.314
Double model	Intercept	− 11.067	–	1.544	− 7.166	< 0.001	-
	Maximum Polyp Diameter (mm)	0.185	0.120–0.325	0.038	4.918	< 0.001	1.203
	Endometrial Thickness (mm)	0.745	0.600–1.004	0.108	6.867	< 0.001	2.106

**Table 5:** AIC, Residual Deviance, Accuracy, Sensitivity, Specificity, AUC value and Optimal Threshold of Logistic regression models and GAM Model by tenfold Cross-Validation

Model		AIC	Residual deviance	Accuracy	Sensitivity	Specificity	AUC	Optimal threshold
Logistic regression model	Full model	157.03	145.03 (189)	0.841	0.820	0.858	0.896	0.473
	Double model	154.41	148.41 (192)	0.841	0.809	0.868	0.902	0.506
GAM model		148.23	134.2 (187.987)	0.856	0.820	0.887	0.906	0.485

The GAM model was employed to capture the potentially non-linear relationship. It revealed that significant effects between maximum polyp diameter (p < 0.01), endometrial thickness (p < 0.01), and IUBP duration. The performance of the model was proved by AUC of 0.906 with tenfold cross-validation (Table 5). When the endometrial thickness was less than 10 mm or the maximum polyp diameter was less than 10 mm, the model predicted relatively high odds of stopping bleeding within 3 months. In contrast, once the endometrial thickness exceeded 15 mm or the max polyp diameter exceeded 20 mm, patients are more likely to have bleeding prolonged beyond 3 months (Fig. 3-D, E). To further substantiated the performance of our predictive models, we employed the bootstrap approach on the full dataset in addition to cross-validation. This method was utilized to rigorously assess the robustness of the area under the ROC curve (AUC) and to compute its corresponding 95% confidence intervals, thereby enhancing the reliability and scientific validity of our findings. The AUC estimates for the different models were as follows: Full Model AUC = 0.912 (95% CI: 0.877–0.952), Double Model AUC = 0.906 (95% CI: 0.870–0.944), and GAM Model AUC = 0.922 (95% CI: 0.884–0.954).

## Discussion

Endometrial polyp is the common benign endometrial lesion, primarily characterized by the excessive proliferation of local endometrial glands and stroma [1]. It contains significantly higher concentrations of estrogen receptors and progesterone receptors in the glandular epithelium compared to normal endometrial epithelium [12]. Hysteroscopic polypectomy allows for the complete removal of the polyps under direct visualization [3]. However, to cope with its high recurrence rate, appropriate endometrial management with progestins after surgery is necessary, especially for the non-functional polyps with hormone-related [13]. According to a study by Sun et al., the rate of adverse effects (including liver dysfunction, venous thrombosis, and breast cancer risk) was significantly lower (RR = 0.17, p < 0.0001) in patients treated with hysteroscopic polypectomy plus an LNG-IUS than in an oral progestin group [14]. This finding provides a basis for expanding the therapeutic role of this device beyond contraception. We identified 4 recurrences (2.05%) within one year. This result is comparable to the before findings [15, 16]. Notably, among the recurrent cases, one presented a slightly hyperechoic area on ultrasound, which was subsequently confirmed by hysteroscopy to be a false diagnosis. By reviewing hysteroscopic images of the other recurrent surgeries (Figure S2 B, C), we found that polyp recurrence was due to incomplete removal during hysteroscopic curettage. This highlights the necessity of completely removing endometrial polyps while protecting the normal endometrium [17]. (Additional file 2_FigureS2).

Considering the increased risks of thrombosis, breast disease, and metabolic disorders associated with long-term combined oral contraceptive use [18], clinicians often prefer the LNG-IUS for endometrial management in patients over 40 after hysteroscopic polypectomy. Therefore, to compare the side-effect profiles of the LNG-IUS between these two clinically distinct age groups, we employed the 40-year threshold. This cutoff ensured statistical robustness, created balanced cohorts, and provided a safe, actionable threshold for patient counseling. Our study indicated that patients aged ≥ 40 years were more likely to develop amenorrhea after LNG-IUS insertion. This finding aligns with existing studies suggesting that LNG-IUS-induced amenorrhea is closely linked to the patient's baseline menstrual pattern and age-associated variations in endometrial responsiveness to hormones [19–21]. LNG-IUS reduces menstrual bleeding by inhibiting endometrial growth and decreasing angiogenesis, with some patients even experiencing amenorrhea [22]. To ensure that amenorrhea was attributable to the LNG-IUS rather than natural menopause, patients over 40 who developed amenorrhea underwent endocrine examination. This step was crucial not only for the scientific rigor of our cohort but also for clinical practice, as the risk of polyp recurrence declines after menopause and device removal may be considered. Conversely, we observed that patients under 40 years old were more likely to experience pelvic pain after LNG-IUS placement, which may be related to differences in the daily amount of exercise or frequency of sexual activity between age groups. However, previous studies have also suggested that the occurrence of pelvic pain with LNG-IUS placement may be associated with the size of the follicle development during the female menstrual cycle, as well as with migration of the intrauterine device [23–25]. It should be noted that approximately one-fifth of cases reported coital bleeding without cervical lesions, the most likely reason was that these cases likely mistook spotting caused with LNG-IUS placement for coital bleeding.

Suvisaari et al. found that patients receiving the LNG-IUS during induced abortion experienced reduced rates of irregular bleeding and an increased rate of amenorrhea. This effect is likely owing to the extensive removal of the endometrial tissue during the procedure [26]. Our study indicated that insertion LNG-IUS at polypectomy did not compromise its efficacy in preventing polyp recurrence and increase risk of adverse reactions. It should be noted that this result has to some extent addressed the concerns of surgeon about the risk of device detachment during simultaneous LNG-IUS insertion and surgery for polyps. Meanwhile, it avoids the need for a secondary procedure reducing the patient discomfort and socio-economic expenditure. It allows direct visualization of LNG-IUS positioning under hysteroscopy, enabling immediate adjustments if necessary [27]. Furthermore, none of cases who received LNG-IUS insertion at surgery was found to have malignant transformation of polyps in postoperative pathological examination. This can be attributed to the low probability of malignant transformation of endometrial polyps in premenopausal women [28]. Additionally, all hysteroscopic surgeries in this study were performed by experienced physicians, whose experience might have played a great role in distinguishing benign and malignant lesions during operation.

Irregular bleeding state with LNG-IUS placement is caused by the persistent intrauterine action of levonorgestrel, which affects by inhibiting endometrial proliferation, inducing decidualization of endometrial stromal cells, causing atrophy of glands and surface epithelium [29], reducing blood flow, and increasing expression of VEGF and upregulation of 17β-hydroxysteroid dehydrogenase type 2 (17βHSD2), enhancing vascular permeability and fragility [30, 31]. It results in unstable endometrial shedding, thereby causing irregular spotting. Until a new menstrual pattern emerges owing to the endometrium gradually adapts, it presents with reduction in spotting or amenorrhea. Therefore, we believe that IUBP is a process of transition from the patient's previous menstrual pattern to a new menstrual pattern. However, due to individual variations in endometrial responsiveness to hormonal changes, the duration of irregular bleeding varies among patients [20, 22]. Therefore, the primary objective of our study was to develop a predictive model for the duration of IUBP to make it predictable through preoperative assessment, thereby enabling personalized patient counseling, improving compliance, and ultimately ensuring the long-term effectiveness of LNG-IUS in preventing polyp recurrence.

This study represented the first evaluation of risk factors associated with IUBP duration in a cohort of patients with endometrial ploy who underwent hysteroscopic endometrial polypectomy combined with LNG-IUS placement. Endometrial thickness and maximum polyp diameter became the core independent variables with statistically significant differences between the two groups of IUBP duration. The models enhanced predictive performance but also demonstrated that bigger polyp or thicker endometrium was associated with a disproportionately higher risk of extended bleeding duration. Full-data logistic regression model analysis validated strongly these two factors as statistically significant predictors. Introduced the interaction term of the significant variables, the interaction term was not shown to be significant, the overall calibration showed limited improvement (Additional file 1_TableS2, Additional file 2_FigureS3). It implied that the effects of these two anatomical factors may be largely independent. Double model had a good level of fit and discrimination; as the more parsimonious model, it can be chosen depending on clinical utility value. GAM model revealed significant non-linear effects for maximum polyp diameter and endometrial thickness and performed slightly better than logistic regression models (Additional file 2_FigureS4).

Clinically, these results were highly significant. First, these two significant preoperative factors are related to the anatomical characteristics of the polyp. Although these characteristics are inherent to the disease, preoperative or intraoperative interventions (such as preoperative mifepristone [32] or GnRH-a therapy or intraoperative curettage) could be attempted to modify them. This may shorten the duration of IUBP, thereby improving patients' postoperative quality of life, satisfaction, and compliance. Second, the superior performance of Double model highlighted the importance of reducing model complexity, providing clinicians with an intuitive framework for identifying key predictors.

## Limitation

As a retrospective study, our research is inherently subject to selection, recall, and information biases owing to its reliance on medical records and self-reported data from telephone interviews. Due to the long study time span and consequent recall bias, we could not reliably collect patient characteristics that relied on preoperative recall—such as BMI and comorbidities. Furthermore, the exclusion of patients who requested removal (e.g., for bleeding intolerance) means our findings on continuation rates and tolerability may overestimate real-world effectiveness. As a single-center study, our findings require validation through future multi-center prospective trials. Moreover, while the sample size was adequate for our primary goal of predictive modeling, it limits the power to detect significant differences in rare adverse events, such as polyp recurrence. Furthermore, the pragmatic use of different polypectomy techniques (e.g., mechanical vs. electrosurgical) may introduce a source of bias for result, although current evidence suggests the choice of technique has no significant impact on recurrence rates [2]. Future prospective studies should include all patients from the point of device insertion to ensure generalizability; such studies, integrating larger multi-center datasets, should also incorporate a broader range of predictors, including surgical techniques, hormonal baseline, BMI, comorbidities, and assessments of psychological changes and quality of life, to enhance the predictive performance and clinical applicability of the models.

## Conclusion

Hysteroscopic polypectomy combined with LNG-IUS insertion was an effective measure to treat endometrial polyps. LNG-IUS intraoperative placement did not increase the incidence of adverse reactions. Preoperative endometrial thickness and maximum polyp diameter play a key role in valuable prediction for the duration of irregular uterine bleeding after LNG-IUS insertion.

## Supplementary Information

Additional file 1

Additional file 2

Additional file 3

## Data Availability

The datasets generated and analyzed during this study include anonymized patient follow-up records and transvaginal ultrasound data, which were compiled into structured Excel files for analysis. Due to patient privacy protections and institutional data security policies, these raw datasets are not publicly available. De-identified data (with all personal information removed) may be shared upon reasonable request to the corresponding author, subject to approval by the Ethics Committee of the First Affiliated Hospital of Chongqing Medical University. Researchers seeking access must submit a formal proposal outlining the purpose of the request and agree to comply with confidentiality agreements.

## Abbreviations

LNG-IUS: Levonorgestrel-releasing intrauterine system
IUBP: Irregular uterine bleeding pattern
GAM: Generalized additive model
TVUS: Transvaginal ultrasound
