# Supplementary material for: Prediction of menstrual patterns and analysis of adverse effects for hysteroscopic endometrial polypectomy combined with LNG-IUS treatment: a single-center retrospective cohort study
Source: Eur J Med Res. 2026 Jan 5;31:195. doi: 10.1186/s40001-025-03776-w (PMC12870506; doi:10.1186/s40001-025-03776-w)
Supplement: Supplementary file 2 — Additional file 2 [file 40001_2025_3776_MOESM2_ESM.pptx]

## Slide 1
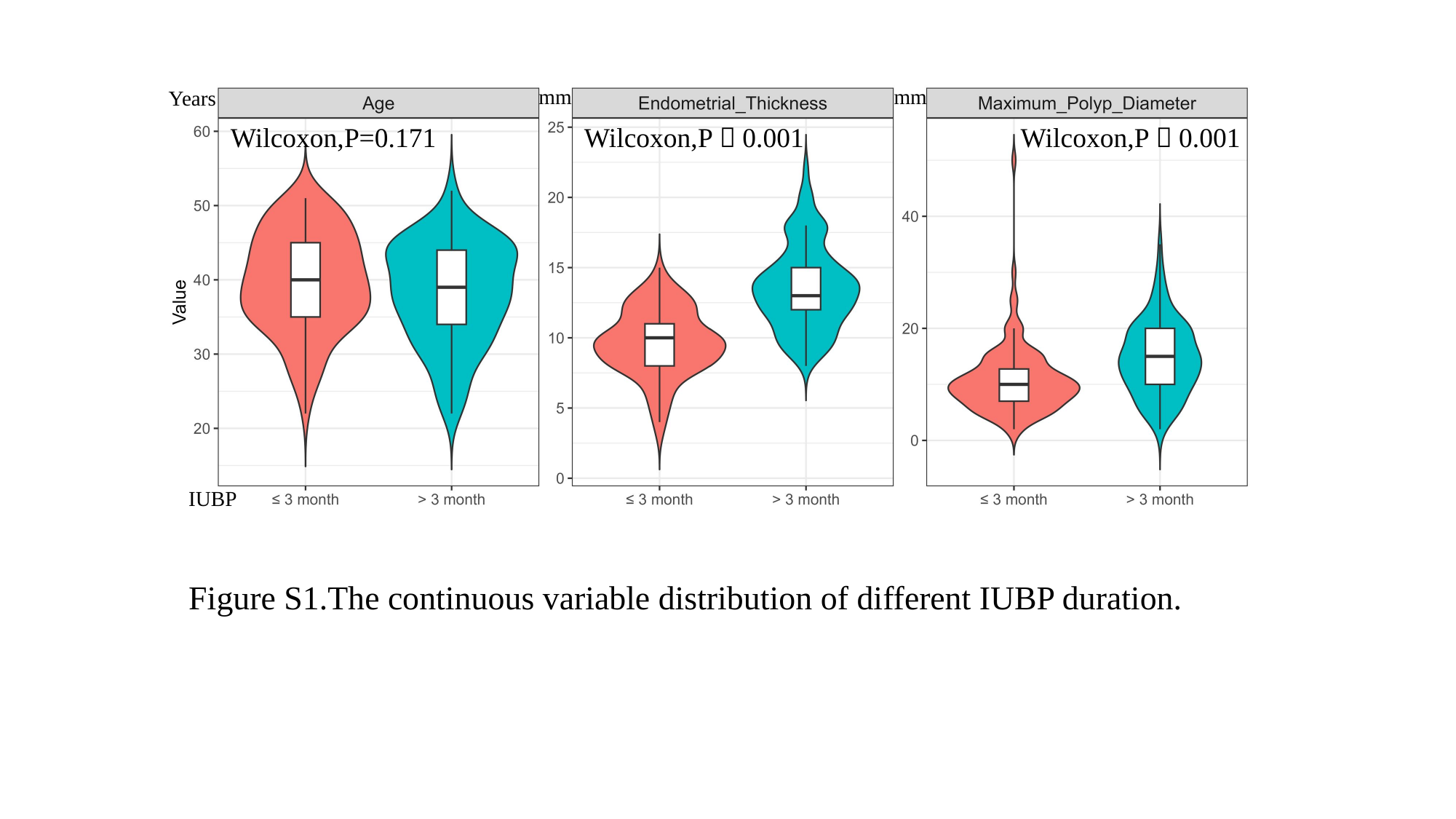

mm
mm
Years
Wilcoxon,P=0.171
Wilcoxon,P＜0.001
Wilcoxon,P＜0.001
IUBP
Figure S1.The continuous variable distribution of different IUBP duration.

## Slide 2
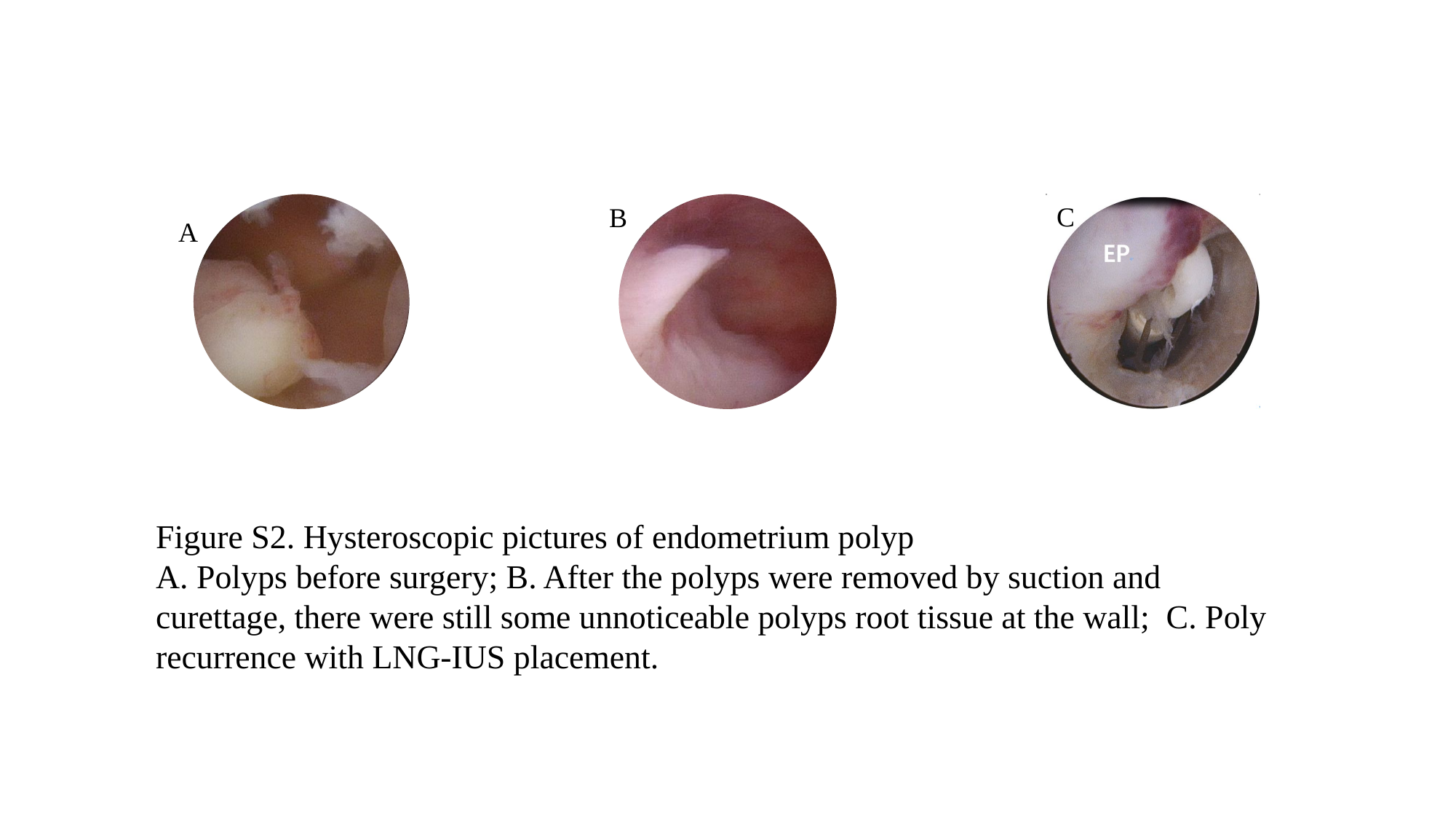

C
B
A
Figure S2. Hysteroscopic pictures of endometrium polyp
A. Polyps before surgery; B. After the polyps were removed by suction and curettage, there were still some unnoticeable polyps root tissue at the wall; C. Poly recurrence with LNG-IUS placement.

## Slide 3
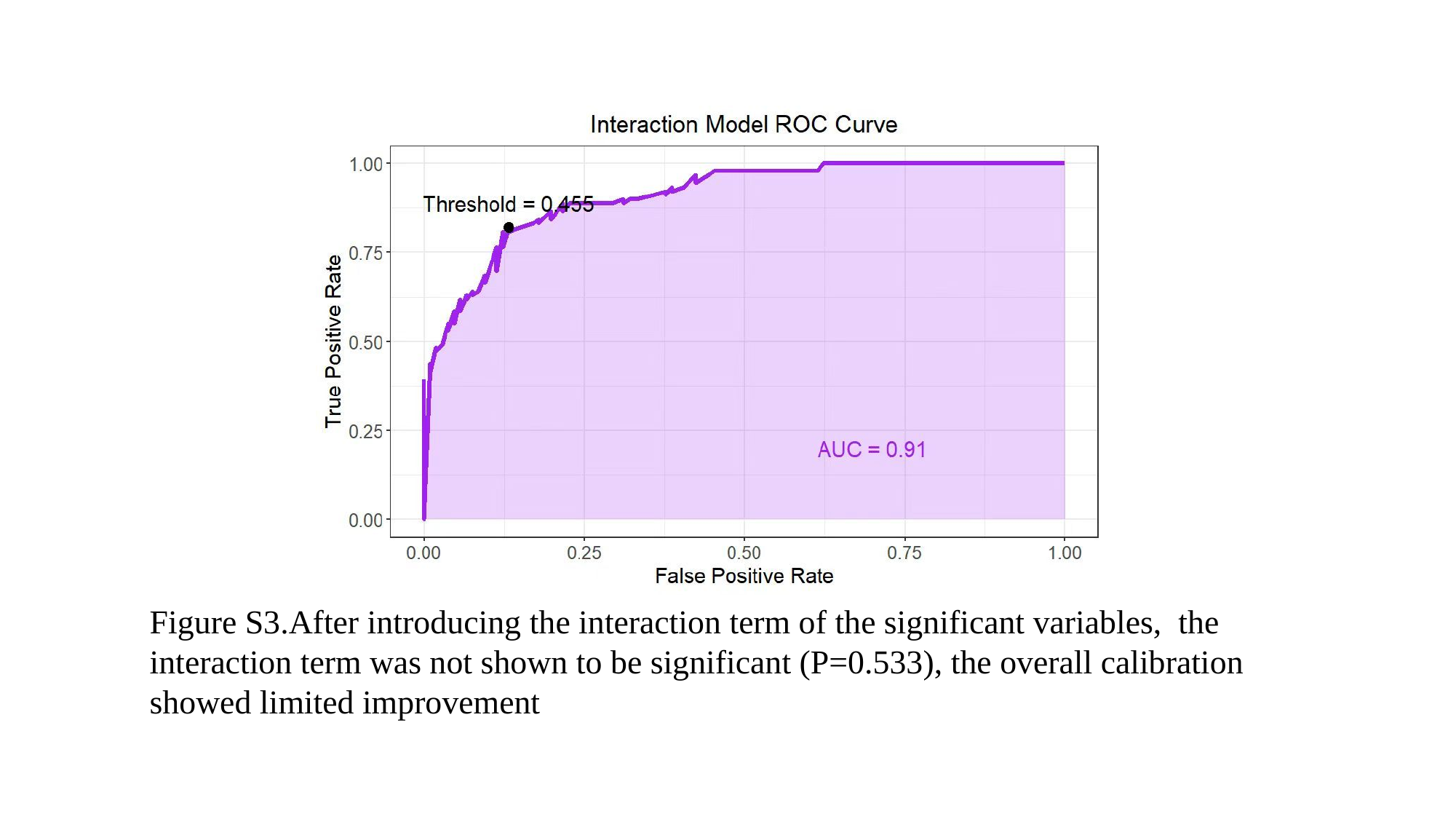

Figure S3.After introducing the interaction term of the significant variables, the interaction term was not shown to be significant (P=0.533), the overall calibration showed limited improvement

## Slide 4
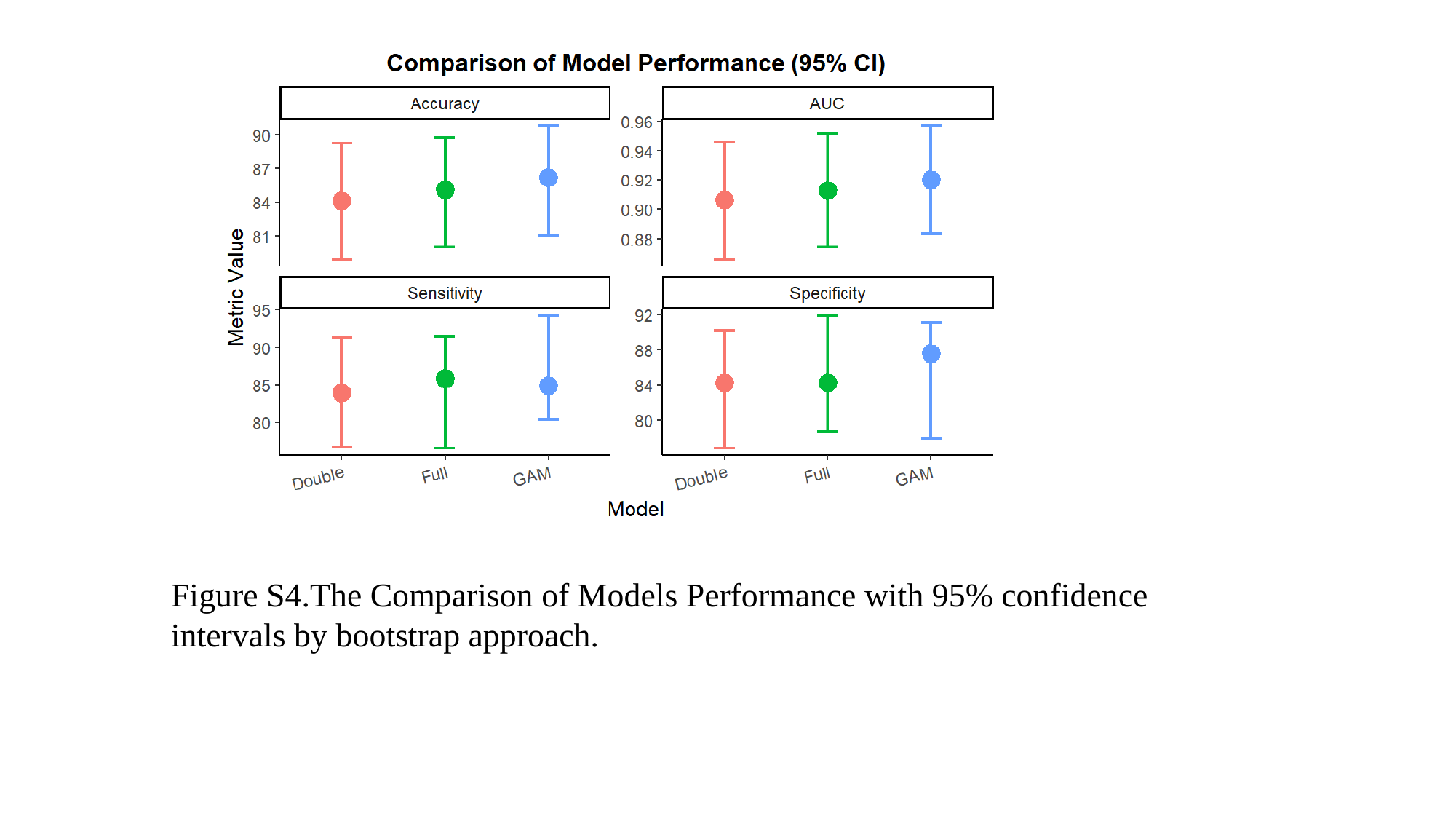

Figure S4.The Comparison of Models Performance with 95% confidence intervals by bootstrap approach.

## Slide 5
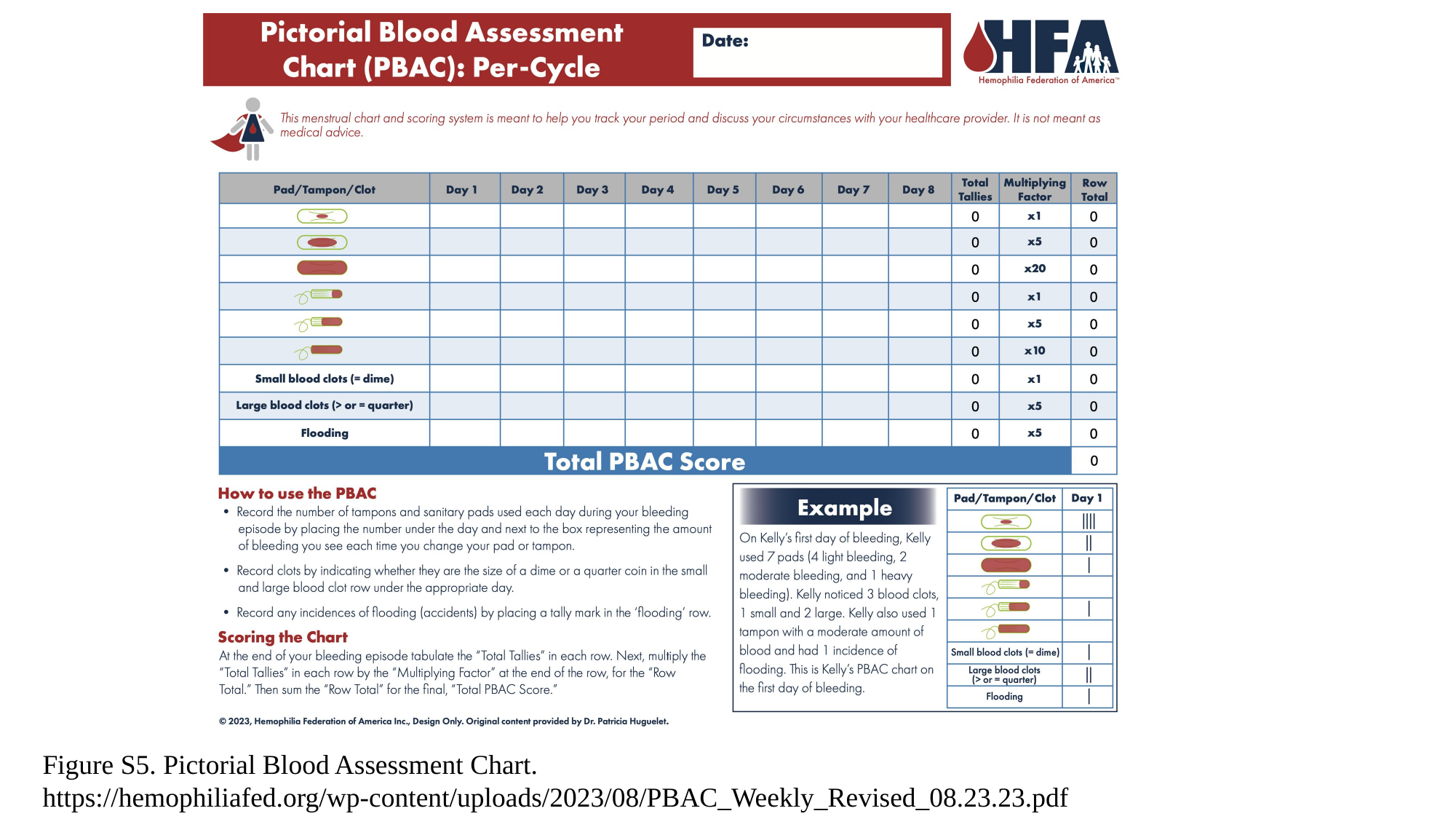

Figure S5. Pictorial Blood Assessment Chart.
https://hemophiliafed.org/wp-content/uploads/2023/08/PBAC_Weekly_Revised_08.23.23.pdf
